# Preparation of TiO_2_ Nanotubes Loaded on Polyurethane Membrane and Research on Their Photocatalytic Properties

**DOI:** 10.1155/2017/9629532

**Published:** 2017-01-17

**Authors:** Longli Lin, Qijun Wu, Xun Gong, Yu Zhang

**Affiliations:** ^1^College of Chemical Engineering, Guizhou University of Engineering Science, Bijie 551700, China; ^2^School of Chemical Engineering, Guizhou Institute of Technology, Guiyang 550003, China; ^3^School of Light Industry, Guizhou Institute of Technology, Guiyang 550003, China

## Abstract

To solve the problem of separation and recovery of photocatalyst in water, the modification of TiO_2_ was studied as well as its immobilization and photocatalytic properties. To improve surface properties, TiO_2_ nanotubes were synthesized by a hydrothermal method and silylated by silane coupling agents to introduce a certain functional group. Supported on polyurethane (PU) membrane, TiO_2_ nanotubes were prepared to produce immobilized PU/TiO_2_. Catalysts were characterized and identified by means of Fourier-transform infrared spectroscopy (FTIR), attenuated total reflectance Fourier-transform infrared spectroscopy (ATR-FTIR), and scanning electron microscopy (SEM). Results showed that silylated TiO_2_ nanotubes were well grafted on the surface of the activated PU membrane. With a 300 W high pressure mercury lamp as light source, the photocatalytic activity and stability of immobilized PU/TiO_2_ were investigated with degrading methyl orange. It was showed that the target is degraded by immobilized PU/TiO_2_ with high activation and the catalytic performance is stable for a long time if catalyst is washed with ethanol.

## 1. Introduction

Considered as the highest potential on the development of environmentally friendly photocatalyst, TiO_2_ was focused on regarding its mechanism and application of pollutants removal from water. Electron-hole pairs would be consistently generated from semiconducting TiO_2_ under irradiation. When electron-hole pairs were trapped by the contaminant adsorption on the surface of TiO_2_, the alternative reaction occurred. Due to the achievement of electron-hole pairs, such dissolved transition metal ions in water as Tl (I) [1], Cr (VI) [2], Hg (II) [3], and Cu (II) [4–6] were reduced. However, such organic pollutants as the most refractory ones were oxidized (e.g., pesticides, herbicides, dyes, surfactants, and electroplating additives) [7], in addition to salicylic acid [8], EDTA [9], phenols [10], and azo dyes [11] and so on.

Currently, powdered TiO_2_ photocatalysis was generally used to remove contaminants. However, powdered TiO_2_ is difficult to be separated and recycled from the aqueous solution [12] and its activity will be reduced since it is easy to reunite together in water. For the reasons above, immobilized TiO_2_ attracted widespread attention in the field of its application. Such porous materials as porous aluminum oxide [13], silicon oxide [14], molecular sieve [15], and active carbon [16] were often used as the carrier of TiO_2_. However, the problem of catalyst separation and recovery from the reaction medium still exists since the carriers above are in a form of particles as themselves. For the reasons above, new forms of TiO_2_ immobilized are highly to be gotten for its application of TiO_2_. In this study, the immobilized PU/TiO_2_ was prepared with organic plastic (polyurethane membrane), and its activity and stability were tested as well.

## 2. Materials and Methods

### 2.1. The Main Reagents and Instruments

The main reagents and instruments are as follows: TiO_2_ (Degussa, Germany), methyl orange (Aladdin, China), toluene-2,4-diisocyanate (PDI, China), PU membrane (0.1 mm), *γ*-ammonia propyl triethoxy silane coupling agent (KH-550, China), a 300 W mercury lamp (main wavelength 365 nm, Shanghai Yaming Lighting Co., Led., China), multifunctional photochemical reaction (SGY-II, China), Fourier-transform infrared spectroscopy (EQUINOX55S, Germany), attenuated total reflectance Fourier-transform infrared spectroscopy (Nicolet 5700, Thermo ESI, USA), scanning electron microscope (Ultra 55, ZEISS, Germany), Gemini V specific surface area instrument (Mike, US), and 722 UV-vis spectrophotometer (Shanghai, China).

### 2.2. Preparation of Immobilized PU/TiO_2_

#### 2.2.1. Preparation of TiO_2_ Nanotubes

TiO_2_ nanotubes were prepared with mixed crystal P25 TiO_2_ as raw material. Average particle size of crystal P25 TiO_2_ is 21 nm and specific surface area is 59.3 m^2^/g. The ratio for anatase to rutile is about 80 : 20 (mass ratio). Hydrothermal synthesis method was applied to the preparation of TiO_2_ nanotubes [5].

#### 2.2.2. PU Membrane Pretreatment and Surface Activation

PU membrane was cut into 1.0 cm × 1.5 cm. To remove organic materials from its surface, PU membrane was washed successively in ethanol and toluene solution with ultrasonic for 15 mins. Then, PU membrane was dried for 15 hours in vacuum under 40°C and was on standby for next test.

Mix a 20 mL toluene solution and 2 mL PDI with 0.5 mL triethylamine (dry before use) in a 150 mL three-neck flask and then add three pieces of pretreated PU membranes (1.0 cm × 1.5 cm) with magnetic stirring. With nitrogen protection, mixture was heated to the temperature of 60°C and kept at the constant temperature for 1 h so as to fully introduce isocyanate groups on the surface [17]. After the reaction completed, the activated PU membranes (PU-NCO) were eluted several times by dehydrated toluene and then were placed in a vacuum oven and dried for 12 hours at the temperature of 40°C.

#### 2.2.3. TiO_2_ Nanotubes Treated by Silanization

Take 0.4 g TiO_2_ nanotubes into a three-mouth flask containing 40 mL toluene. After 30 mins of ultrasonic dispersion, 1.47 mL KH-550 was add to the three-mouth flask as well as 2 drops of triethylamine. Then it was heated and mixed for 6 hours at the temperature of 60–65°C. After the reaction finished, 120 mL of methanol was added to remove the silane coupling agent molecules residue. Then, sediment was filtered out, washed with methanol, deionized water, and acetone in turn, and then dried for 12 h in vacuum at the temperature of 80°C, and it was on standby for next test [18].

#### 2.2.4. The Preparation for Immobilized PU/TiO_2_

After 6.758 g potassium persulfate was dissolved in a 50 mL volumetric flask, 0.5 mol/L potassium persulfate solution was obtained. Take 0.2 g silylated TiO_2_ nanotubes and pour them into a flask containing 100 mL toluene. After being dispersed by ultrasound for 10 mins, activated PU membrane was added. Then, nitrogen was inlet for protection. It was heated and stirred from the beginning room temperature to 60–65°C. During the process of heat, 5 mL of 0.5 mol/L potassium persulfate solution was slowly dripped into it. After 6 h dark reaction, solution was cooled to room temperature. Sediment was filtered out and repeatedly washed with ethanol. After being dried with vacuum, immobilized PU/TiO_2_ was obtained.

### 2.3. Photocatalytic Devices

**Figure 1:**
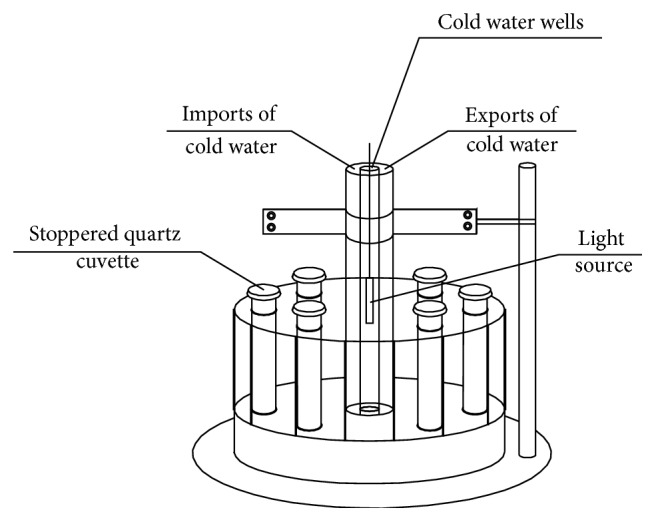
Rotating photochemical reactor schemes.

Photocatalytic reactions were carried out in a roaring photochemical reactor (Figure 1). A 300 W mercury lamp was placed in quartz cooling well. Outside the cooled well were six 50 mL quartz colorimetric tubes, in which were 25 mL 8.00 mg/L methyl orange solution and the immobilized PU/TiO_2_, stirred by a magnetic stirrer. The temperature of the photoreactor was regulated at 26 ± 0.5°C by a circulating water system. The average irradiation intensity in solutions was about 8 MW/cm^2^.

### 2.4. Analytic Determination of Methyl Orange

The photocatalytic activity and stability of immobilized PU/TiO_2_ were evaluated by measuring the photocatalytic degradation efficiency of methyl orange. Stabilization time of the lamp was 1 min. Samples were taken at intervals of 5 mins; concentration of methyl orange was analyzed at 465 nm wavelength by a 722 UV-vis spectrophotometer (thickness of color plate is 1.0 cm). The degradation efficiency for methyl orange can be calculated by the following equation:

(1) $$\begin{array}{c} \eta =\frac{C_{0}-C_{t}}{C_{0}}\times 100\%, \end{array}$$

where *η* is moment degradation efficiency and *C*_0_ and *C*_*t*_ are the initial concentration and the moment concentration of methyl orange in mg/L, respectively.

## 3. Results and Discussion

### 3.1. Structural Characterization

#### 3.1.1. Characterization Analysis for TiO_2_ Nanotubes

FTIR and BET were applied to characterize the prepared TiO_2_ nanotubes. The result showed that the majority of substance in the TiO_2_ nanotubes is a composite of anatase rich in hydroxyl [5], with a small amount of rutile. The BET specific surface area of TiO_2_ nanotubes is 96.7 m^2^/g, which is larger than the BET specific surface area of the raw TiO_2_ nanoparticles (59.3 m^2^/g). The characters proved that the prepared TiO_2_ nanotubes were showed with stronger adsorption ability. The material was theoretically in good photocatalytic activity.

#### 3.1.2. Characterization Analysis for TiO_2_ Nanotubes Treated by Silanization

**Figure 2:**
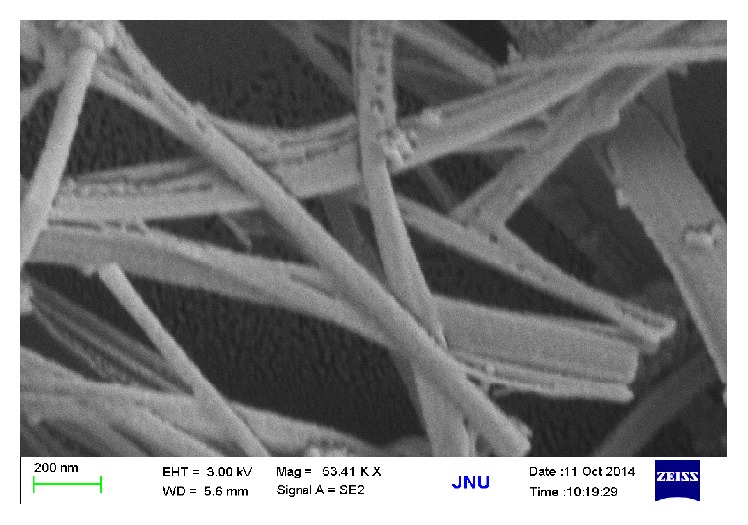
SEM spectrum of TiO_2_ nanotubes treated by silanization.

**Figure 3:**
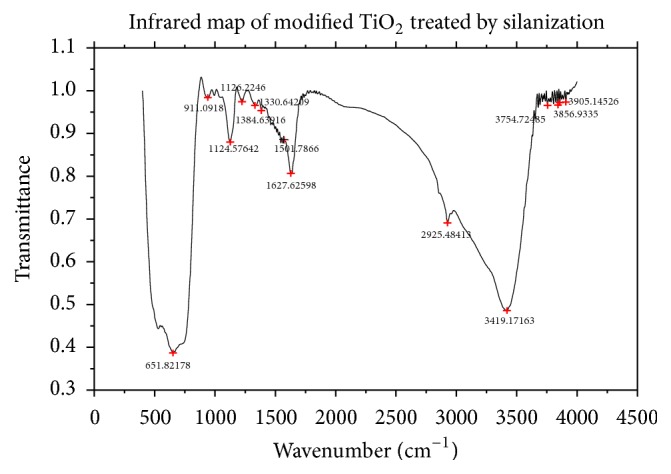
FTIR spectrum of TiO_2_ nanotubes treated by silanization.

TiO_2_ nanotubes treated by silanization were characterized by SEM (Figure 2) and FTIR (Figure 3). A hollow tubular structure was showed with the nanotubes treated by silanization (Figure 2). The absorption peak, Ti-O-Si keys, was found at 911 cm^−1^ of the sample (Figure 3), generated by the condensation of hydrolyzed silane coupling agent conjunct with hydroxyl groups on the surface of the nanometer TiO2 [19]. The absorption peak at 1126 cm^−1^, Si-O-Si keys, displays a condensation effect which occurs in the silanols hydrolyzed by silane coupling agents. Thus combination phenomenon was formed among silanols, silanol, and TiO_2_, which is beneficial to the formation of a multilayer structure on the surface of the carriers and avoids its oxidization by photocatalysis. Absorption peak of interval range was similar to that of amino N-H key for the reason that there is a great quantity of free hydroxyl on the surface of the TiO_2_. The absorption peak at 1501 cm^−1^ proved the existence of N-H keys, but neither 3419 cm^−1^ nor 1627 cm^−1^. Because the interaction between silanol and hydroxyl is on the surface of TiO_2_, the vibration deformation of primary amine was transferred towards low wave area [20]. 2925 cm^−1^ is the stretching vibration peak of methylene. The characteristic peaks showed that specific functional groups were grafted on the surface of nano TiO_2_ through silanization. Modified TiO_2_ nanotube was obtained for grafting ammonia propyl siloxane on the surface.

#### 3.1.3. Characterization of the PU Membrane

**Figure 4:**
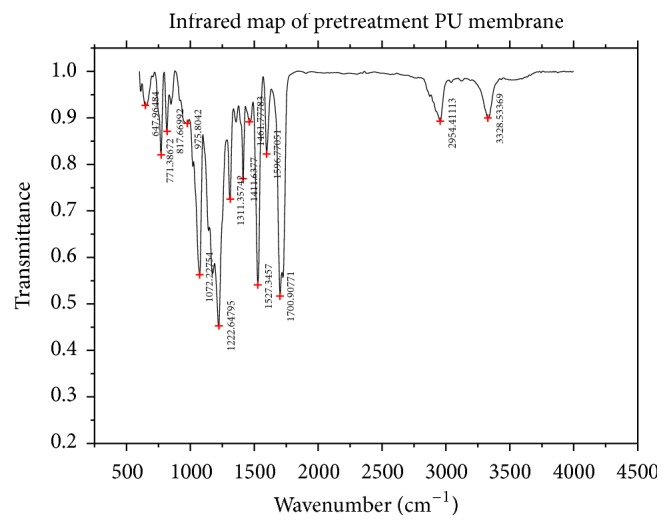
ATR-FTIR spectrum of pretreatment PU membrane.

**Figure 5:**
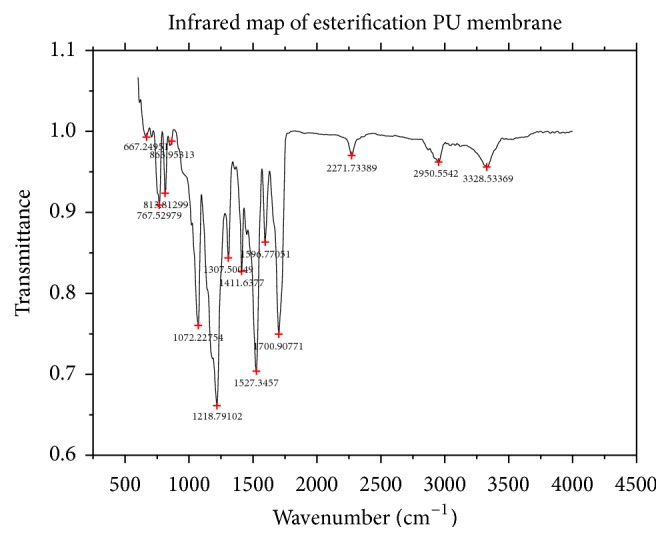
ATR-FTIR spectrum of esterification PU membrane.

ATR-FTIR spectrums of pretreatment PU membrane and esterification PU membrane were shown as follows (Figures 4 and 5). No function reaction group was found on the pretreatment PU membrane surface (Figure 4). Free isocyanate groups were formed with the excess PDI after being catalyzed with triethylamine to react on the surface of PU membrane. To compare Figures 4 and 5, one obviously characteristic peak of isocyanate was found with PU-NCO film at 2271 cm^−1^ [21]. Results showed that isocyanate groups (-NCO) were successfully introduced on the surface of PU membrane.

#### 3.1.4. Characterization of Immobilized PU/TiO_2_

**Figure 6:**
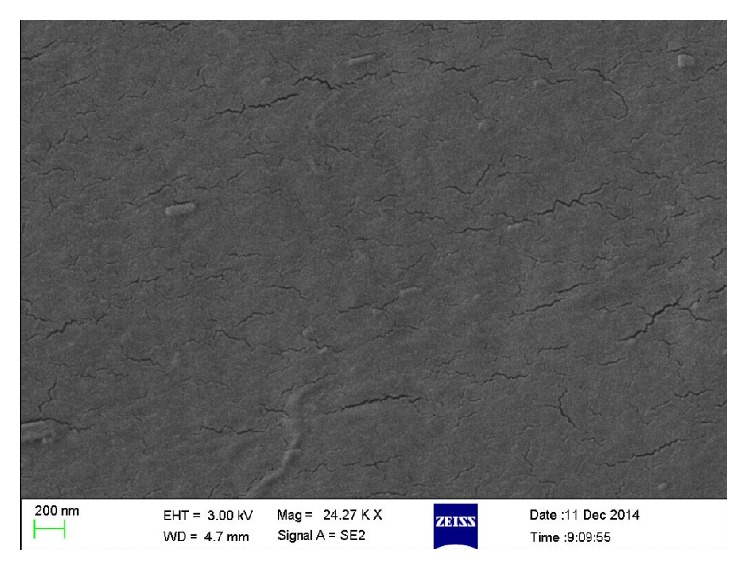
SEM spectrum of esterification PU membrane.

**Figure 7:**
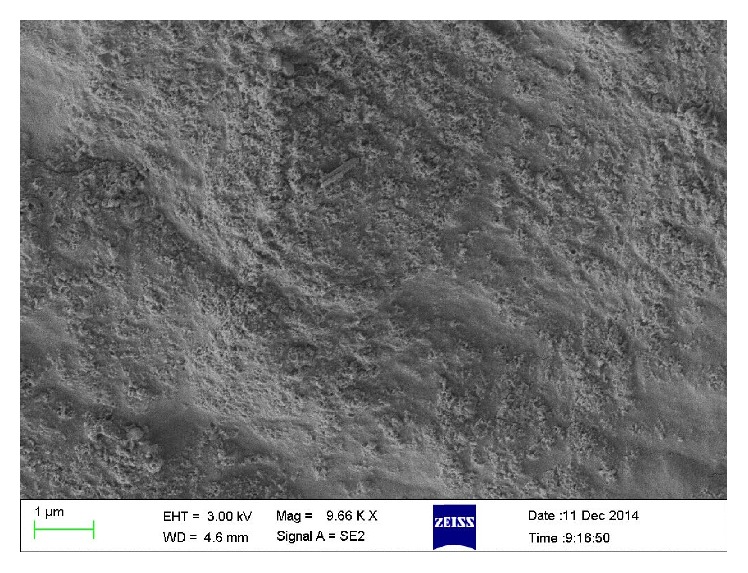
SEM spectrum of immobilized PU/TiO_2_.

SEM spectrums of esterification PU membrane and immobilized PU/TiO_2_ were shown in Figures 6 and 7. Results showed that the PU membrane surface was bright and clean after activation treatment. The surface of PU membrane is coarse after being covered with multilayer TiO_2_ nanotubes. The difference above is reasoned by the link between the bottom of the nanotubes and PU membrane through surface grafting. The hydrolysis of silane coupling agents and hydroxyl groups on the surface of the upper nanometer TiO_2_ condense into Ti-O-Si keys. Then, TiO_2_ nanotubes graft together and form a multilayer mesh structure, although the distribution is not uniform.

**Figure 8:**
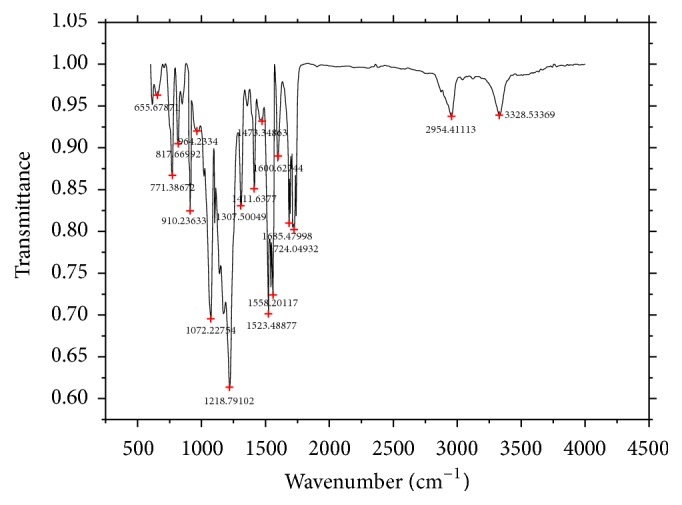
ATR-FTIR spectrum of immobilized PU/TiO_2_.

ATR-FTIR spectrum of immobilized PU/TiO_2_ was shown in (Figure 8). It can be seen that amide carbonyl stretching vibration absorption occurred at 1685 cm^−1^, and “amide II peak” (C-N-H bending vibration) [22] occurred at 1558 cm^−1^. The result proved that grafting reaction occurred between activated PU membrane (PU-NCO) and primary amines of nanotube surface. Then, secondary amides were formed. It was shown that a key absorption peak at the 910 cm^−1^ is Ti-O-Si. Primary amines on the surface of the modified TiO_2_ nanotubes reacted with isocyanate group, so that the TiO_2_ nanotubes load on the surface of PU membrane. In summary, TiO_2_ nanotubes successfully graft on the surface of PU membrane.

### 3.2. Photocatalytic Activity for the Immobilized PU/TiO_2_

**Figure 9:**
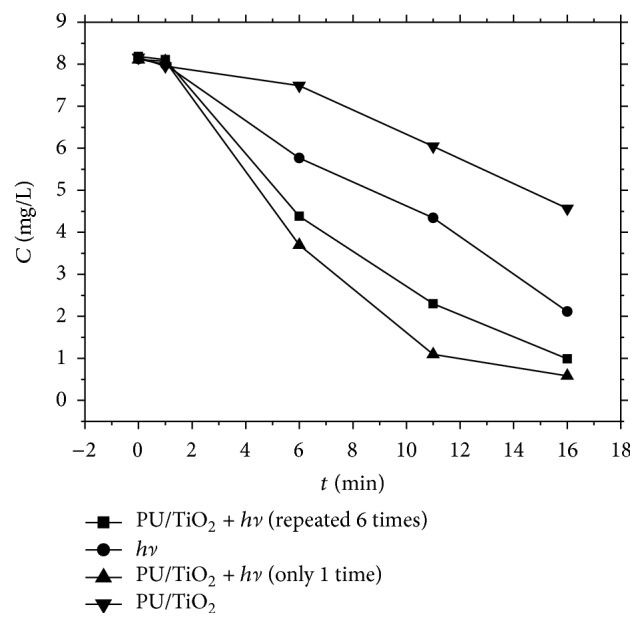
Degradation of methyl orange in different conditions.

As to methyl orange degradation, the photocatalytic activity of immobilized PU/TiO_2_ was showed as follows (Figure 9). After photolysis and dark adsorption experiments were performed, results were depicted as well (Figure 9).

Before the beginning of photolysis, methyl orange solution was accurately measured and add to five 50 mL quartz tubes. Then tubes were put into the photochemical rotating reactor to carry out the photolysis with a 300 W ultraviolet lamp.

In order to ensure establishment of adsorption/desorption equilibrium, dark adsorption was carried out at the same time. 2 pieces of 1.0 cm × 1.5 cm immobilized PU/TiO_2_ were add to per quartz tube; then quartz tube was put into the photochemical rotating reactor. After 5 mins dark adsorption, the photocatalytic degradation reaction was carried out with a 300 W lamp.

Results of dark adsorption showed that concentration of methyl orange is decreasing as time went on. The decreasing proved that methyl orange was effectively adsorbed by immobilized PU/TiO_2_. Results of photolysis showed that the photolysis reaction was directly induced by methyl orange. The results of photocatalytic reaction indicated that the degradation rate of methyl orange was significantly increased. A good photocatalytic activity was shown by immobilized PU/TiO_2_. What is more, the photocatalytic degradation rate was still found higher than that of photolysis although it decreased after being repeatedly used.

### 3.3. Stability of Immobilized PU/TiO_2_

**Figure 10:**
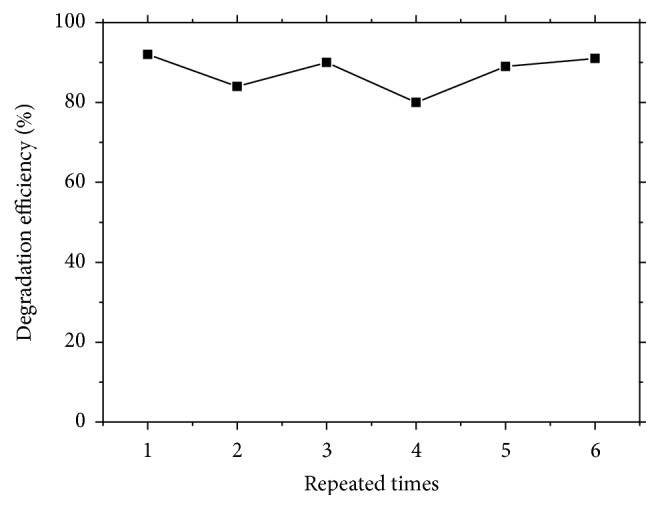
The photodegradation activities of immobilized PU/TiO_2_ after being used repeatedly.

Based on Section 3.2, immobilized PU/TiO_2_ showed a better photocatalytic activity of methyl orange degradation. However, catalytic activity will be declined and catalyst deactivation occurred when immobilized PU/TiO_2_ was repeatedly used without any treatment. In response to this situation, the photocatalytic stability was investigated after methyl orange degraded. The surface of immobilized PU/TiO_2_ was repeatedly washed with ethanol after being used each time (Figure 10). Results showed that the effects of photocatalytic degradation are similar to each other. Photocatalytic degradation was found with a high activity even when used repeatedly six times. Compared with Figures 9 and 10, it was indicated that catalyst deactivation was not caused by the gradual loss of nanotubes. However, the immobilized PU/TiO_2_ film surface is gradually occupied by the reactants and products. The surface of film was not cleaned after being repeatedly used, resulting in the gradual accumulation of inactivation.

**Figure 11:**
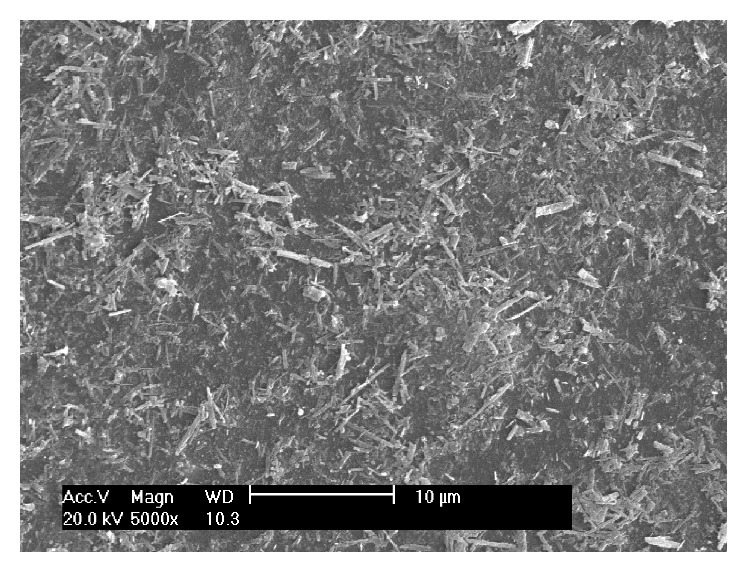
SEM spectrums of immobilized PU/TiO_2_ after being used repeatedly.

The surface morphology of immobilized PU/TiO_2_ was showed as follows after being repeatedly used six times (Figure 11). It was showed that a layer of TiO_2_ nanotube with varied thickness and nonuniform distribution was covered on the surface of PU membrane after being repeatedly used. No significant loss was found with catalyst. It was showed that TiO_2_ nanotube was firmly loaded on PU membrane. Immobilized PU/TiO_2_ still had stable photocatalytic properties even when reused many times.

## 4. Conclusion

Based on the results above, it can be concluded that (1) TiO_2_ nanotubes were prepared by using nanometer TiO_2_ as raw materials. TiO_2_ nanotubes and PU membrane were treated by silanization and surface activation, respectively. Then, activated PU membrane was used as the carrier to prepare immobilized TiO_2_ nanotube and that (2) a higher photocatalytic activity was showed with immobilized PU/TiO_2_ rinsed by ethanol under certain condition. Catalytic activity of immobilized PU/TiO_2_ was stable for a long time even when being repeatedly used many times when washed with ethanol. The separation, recycling, and deactivation problems of the powdered TiO_2_ were well overcame in its application. Immobilized PU/TiO_2_ is a kind of photocatalytic material with a potential application prospect.
